# Role of Angiopoietin-2 in Vascular Physiology and Pathophysiology

**DOI:** 10.3390/cells8050471

**Published:** 2019-05-17

**Authors:** Racheal G. Akwii, Md S. Sajib, Fatema T. Zahra, Constantinos M. Mikelis

**Affiliations:** Department of Pharmaceutical Sciences, School of Pharmacy, Texas Tech University Health Sciences Center, Amarillo, TX 79106, USA; Racheal.Akwii@ttuhsc.edu (R.G.A.); s.sajib@ttuhsc.edu (M.S.S.); fatema.zahra@ttuhsc.edu (F.T.Z.)

**Keywords:** angiopoietin-2, Tie1, Tie2, integrin, angiogenesis, endothelial cells, cancer, inflammation

## Abstract

Angiopoietins 1–4 (Ang1–4) represent an important family of growth factors, whose activities are mediated through the tyrosine kinase receptors, Tie1 and Tie2. The best characterized are angiopoietin-1 (Ang1) and angiopoietin-2 (Ang2). Ang1 is a potent angiogenic growth factor signaling through Tie2, whereas Ang2 was initially identified as a vascular disruptive agent with antagonistic activity through the same receptor. Recent data demonstrates that Ang2 has context-dependent agonist activities. Ang2 plays important roles in physiological processes and the deregulation of its expression is characteristic of several diseases. In this review, we summarize the activity of Ang2 on blood and lymphatic endothelial cells, its significance in human physiology and disease, and provide a current view of the molecular signaling pathways regulated by Ang2 in endothelial cells.

## 1. Introduction

Angiopoietin-2 (Ang2) is a growth factor belonging to the angiopoietin/Tie (tyrosine kinase with Ig and EGF homology domains) signaling pathway, one of the main pathways involved in angiogenesis. Ang2 was identified through a cDNA library screening, shortly after the identification of angiopoietin-1 (Ang1) [1], a potent angiogenic factor, critical for in vivo angiogenesis, with distinct functions from vascular endothelial growth factor (VEGF) [2]. Ang2, a 496 amino acid-long protein, shares ~60% amino acid homology with Ang1 and lacks one of the nine cysteines found in mature Ang1. It has a secretion signal peptide, an NH_2_-terminal coiled-coil domain, and a COOH-terminal fibrinogen-like domain. Unlike Ang1, Ang2 acts in an autocrine manner and its expression is highly regulated. Similar to Ang1, Ang2 binds to the Tie2 receptor with the same binding affinity, inducing its antagonistic role, but does not bind to Tie1 [3]. Ang2 expression is triggered by inflammatory mediators, such as thrombin [4], and conditions, such as hypoxia [5,6] and cancer [7,8]. The aim of this review is to summarize the activity of Ang2 in blood and lymphatic endothelial cells, its different functions in the body under both physiological and pathological conditions, and the main molecular mechanisms identified to date.

## 2. Biological Activity of Ang2 in Endothelial Cells

Although the exact role of Ang2 in angiogenesis has not yet been completely elucidated, due to the constantly increasing body of new data, its role in angiogenesis is now considered context-dependent [9].

### 2.1. Blood Endothelial Cells (BECs)

Under physiological conditions, Ang2 acts as an antagonist in BECs [3]. Ang1 rapidly auto-phosphorylates the Tie2 receptor in endothelial cells, but does not directly promote the growth of cultured endothelial cells [1,10], while Ang2 mainly inhibits Ang1-induced Tie2 phosphorylation [3]. However, later studies demonstrated that Ang2 can also phosphorylate Tie2, though not with the same strength as Ang1, and thus induce endothelial cell functions [11,12]. The agonistic versus antagonistic role of Ang2 was further determined by the presence of Tie1. In inflamed endothelium, Tie1 cleavage led to the switch of the Ang2 activity from agonistic, under normal conditions, to antagonistic [13]. In line with this, Ang2 agonistic activity was absent in Tie1-deficient mice [14]. Ang2 overexpression in mouse embryos led to embryonic lethality at embryonic days 9.5 to 10.5 (E9.5–10.5), and the embryos presented features similar or more severe than the ones with Tie2- or Ang1-deficiency [3], demonstrating the antagonistic function of Ang2 on Tie2. Ang2 is not critical for embryonic vascular development, as Ang2-deficient mice were born at expected ratios. However, it seems to regulate angiogenic sprouting and vascular regression in the neonatal eye, where it acts as a Tie2 antagonist [15]. The endothelial destabilizing effect of Ang2 was demonstrated in a three-dimensional co-culture model of endothelial cells and smooth-muscle cells, where the co-cultures were stimulated with Ang1, Ang2, and VEGF. Ang2 disturbed the endothelial cell monolayer integrity by acting through an autocrine loop mechanism, and Ang2-induced endothelial destabilization was rescued by Ang1, VEGF, or soluble Tie2 treatment [16]. The agonistic role of Ang2 in angiogenesis has been demonstrated in different conditions [17,18,19], in the absence of Tie2 [17,20] or Ang1 [11,12] and in pathological conditions [18,19]. During angiogenesis-driven endothelial cell remodeling, Tie2 expression was downregulated in a subpopulation of endothelial cells, while expression of integrin or Ang2 was not affected. Ang2 was reported to bind to αvβ3, αvβ5, and α5β1 integrins of endothelial cells with lesser affinity, in comparison to its binding to Tie2, inducing angiogenesis in a Tie2-independent manner [17]. In vitro, Ang2 overexpression induced endothelial cell migration and tube formation [17]. Although lower Ang2 concentrations ranging from 50 to 400 ng/mL did not induce any notable angiogenic effects, higher Ang2 concentrations (800 ng/mL) induced endothelial cell survival through Tie2, phosphoinositide 3-kinase (PI3K), and Akt activation, denoting a positive effect on angiogenesis [21]. Conditioned media from transfected cells overexpressing Ang2 led to modest induction of cell proliferation and the formation of extensive capillary-like structures of human umbilical vein endothelial cells (HUVECs) cultured on fibrin matrices in a dose-dependent manner and in the absence of Ang1 [11]. Many more studies have demonstrated the angiogenic role of Ang2 in an indirect fashion, through Ang2 inhibition in pathological angiogenesis. In solid tumors, anti-Ang2 therapy resulted in tumor stasis, and in the rat corneal model, Ang2 inhibited VEGF-induced angiogenesis [18]. In a similar approach, Ang2 knockdown in Kaposi’s sarcoma-associated herpesvirus-infected endothelial cells blocked angiogenesis and inhibited macrophage infiltration and tumor growth [19].

### 2.2. Lymphatic Endothelial Cells (LECs)

In LECs, the role of Ang2 in lymphangiogenesis seems to be more agonistic than antagonistic. High dose treatment of Ang2 (800 ng/mL) phosphorylated the Tie2 receptor and promoted LEC proliferation and cell survival [22]. The angiogenic and anti-apoptotic activity of Ang2, through binding to the Tie2 receptor and activation of the downstream Tie2/Akt signaling pathway, has been attributed to the low levels of physical association of Tie2 with Tie1, due to the decreased Tie1 expression in the LECs compared to BECs, implying an inhibitory effect of Tie1 on Ang2-induced Tie2 activation in LECs [23]. This Ang2 effect is also attributed to the absence of the vascular endothelial protein tyrosine phosphatase (VE-PTP) in the lymphatic endothelium, a protein recently found to regulate the agonistic and antagonistic role of Ang2 on the Tie2 receptor. This was further demonstrated in the blood endothelium, where VE-PTP inhibition converted Ang2 into a potent Tie2 activator [24]. Ang2 plays a significant role in lymphatic development in vivo, as Ang2-deficient mice presented generalized lymphatic dysfunction, characterized by chylous ascites and milky fluid within their peritoneal cavity. Though the pups were able to survive through adulthood, they also exhibited signs of subcutaneous edema and the smaller lymphatic vessels exhibited abnormal patterning [15]. The lymphatic vessels of Ang2-deficient mice presented an immature phenotype, with defects on the collecting lymphatic vessels and valves, deficient postnatal remodeling of the dermal lymphatic vasculature, and premature smooth muscle cell recruitment [25]. However, the poor lymphatic patterning upon Ang2 deficiency was reversed by *Ang1* knock-in [15,25]. It is therefore evident that more studies are required for the elucidation of the precise role of Ang2 on the vascular beds of different organs during steady state conditions.

## 3. Role of Ang2 in Physiological Functions

Developmentally, the role of Ang2 is important, as shown from its expression pattern during embryonic development, but is not critical for embryonic survival. Ang2 mRNA was detected mostly in the dorsal aorta and the major aortic branches, exhibited a punctate expression pattern, and was present in endothelium-associated cells, most likely pericytes [3]. Through the LacZ reporter, Ang2 expression was later confirmed in the smooth muscle cells of large arteries and at the major arterial branches. It was not detected in smaller vessels, except from sites of vascular remodeling [15]. During embryonic development, Ang2 is expressed in the developing placenta, with its expression being highest during early gestation [26], and participates in spiral artery remodeling [6]. It was also demonstrated that Ang2 is important for cranial neural crest cell migration, thus for vertebrate head patterning [27].

Schlemm’s canal is a hybrid vessel bearing blood and lymphatic vessel characteristics and deficiencies in its development and functionality can lead to glaucoma. Although Ang2 seems dispensable for Schlemm’s canal development, its function is compensatory to Ang1, as combined Ang1 and Ang2 deficiency exacerbated the phenotype observed in the Ang1-deficient mice [28]. In intrauterine growth retardation (IUGR), Ang2 expression was decreased, which is an indication of a potential involvement in the villous vasculature development [29]. In the normal adult, expression of Ang2 is predominant in sites of vascular remodeling, particularly the ovary, placenta, and uterus [3]. Ang2 is a Weibel-Palade body molecule with a long half-life of more than 18 h and can be secreted within minutes of stimulation by compounds, such as phorbol myristate acetate (PMA), thrombin, and histamine, which demonstrates a role in vascular homeostatic reactions [30].

MicroRNAs (miRs) are important cancer regulators, functioning either as oncogenes or tumor suppressor genes. Similar to the case of VEGF, Ang2 expression is regulated by miRs, more specifically miR-351 [31], and the Ang1/Ang2 ratio determines the biological activity [6]. MiR-150 has also been reported to regulate Ang2 expression and downstream signaling during vascular injury [32]. Based on the Ang2 expression pattern in sites of vascular remodeling during adulthood, such as areas of vascular wound healing, it would be interesting to explore its potential role during the epithelial-to-mesenchymal transition (EMT) process, as it has been shown to occur in the case of cancer (elaborated in the cancer section).

**Figure 1:**
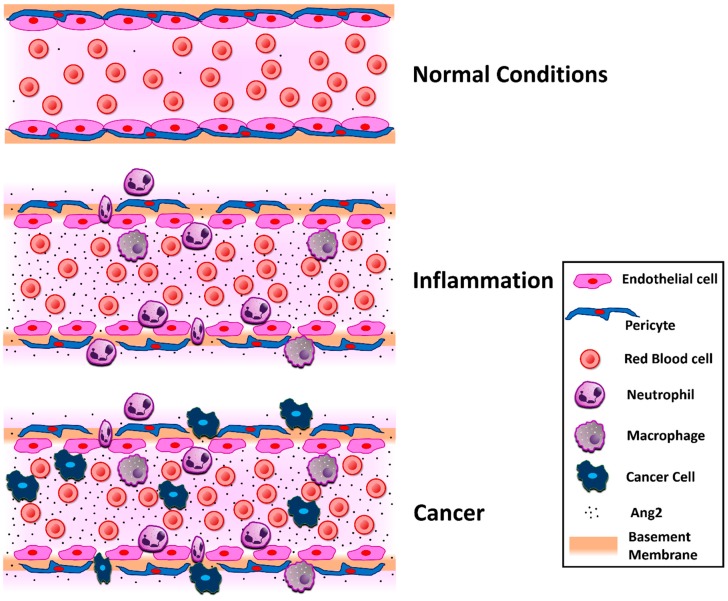
Schematic representation of the Ang2 effect on the vascular bed in normal conditions, inflammation, and cancer. Under normal physiological conditions, Ang2 levels are low, but are upregulated during inflammation or cancer. Ang2 acts on endothelial cells, increasing endothelial permeability and also on the pericytes, causing pericyte detachment from the basement membrane, further inducing vascular leakiness, immune or/and cancer cell trans-endothelial migration, and deterioration of the condition. Ang2 has been proposed as a marker for inflammatory conditions and cancer.

Pericytes are mural cells surrounding the endothelial cells in vascular structures, including capillaries, post-capillary venules, and terminal arterioles. They communicate with the endothelial cells through paracrine signaling and facilitate important functions in vascular physiology, such as blood vessel formation, capillary constriction and dilation, blood-brain barrier maintenance, and regulation of immune cell entry [33,34]. During hyperglycemia or hypoxia, increased Ang2 levels activate Tie2, inducing pericyte detachment from the basement membrane and pericyte migration [34] (Figure 1). Absolute pericyte coverage determines vascular permeability in the blood-brain barrier and moreover, pericytes inhibit the expression of proteins, inducing vascular permeability. Mice that lacked pericytes presented higher Ang2 levels, suggesting that pericytes possibly regulate Ang2 levels, restricting vascular permeability, and thus revealing the importance of Ang2 as a permeability mediator [34,35].

## 4. Role of Ang2 in Disease

Ang2 plays a central role in diseases related to vascular permeability and angiogenesis. Its vascular disrupting properties became evident when Ang2-overexpressing transgenic mice were embryonically lethal due to poorly formed blood vessels [3]. In terms of pathological angiogenesis, the role of Ang2 has been mostly explored in tumor-induced angiogenesis, where its inhibition or overexpression decreased or increased the tumor size and metastatic efficacy, respectively [36,37,38]. The promising results of Ang2 inhibition in cancer have led to ongoing clinical studies [39]. Below, we describe vascular-related pathological conditions, where the role of Ang2 has been mostly defined.

### 4.1. Inflammation

Angiogenesis and inflammation are two correlated conditions [40], and Ang2 has been shown to participate in several cancer-independent inflammatory conditions, such as autoimmune diseases, sepsis, and acute lung injury [41]. It was mentioned earlier that the pro- or anti-angiogenic activity of Ang2 is context-dependent. One of the parameters that define Ang2 activity is the expression of other angiogenic growth factors, such as VEGF. It was reported that Ang2 induced permeability and angiogenesis on the pupillary membrane in the presence of VEGF, as exhibited by the absence of VE-cadherin junctions and the elevated levels of endothelial cell proliferation and migration. In the absence of VEGF, however, Ang2 led to vessel regression and endothelial cell death [42]. Ang2 administration induced edema in a mouse paw edema inflammatory model [43]. Co-administration of submaximal Ang2 and VEGF doses confirmed their additive effect on vascular permeability, although that was not the case in the co-administration of maximal doses, where the increase in paw volume was similar to that observed when either of the growth factors was administered alone [43]. The molecular mechanism for Ang2-induced permeability is also attributed to its interaction with cell junction proteins, such as integrins and VE-cadherin, explained in the relevant section. Ang2 is important for the initiation of the inflammatory response, since Ang2-deficient mice could not initiate an inflammatory response in short-term infection experiments, such as thioglycollate-induced or *Staphylococcus aureus*-induced peritonitis models. Moreover, treatment with recombinant Ang2 evoked an inflammatory response [44]. Serum Ang2 levels have been positively associated with inflammatory biomarkers, such as high-sensitive C-reactive protein and white blood cell count, leading to the notion that Ang2 can be considered an inflammatory marker [45] (Figure 1). In autoimmune diseases, increased angiogenesis is characteristic during the early events. It has been observed that Ang2 is present in inflammatory conditions generated by autoimmune diseases, such as vasculitis [46] and lupus [47]. In systemic lupus erythematosus patients, serum Ang2 levels were increased, indicating that Ang2 contributes to inflammation, permeability, and premature atherosclerosis, and was proposed as a biomarker for disease activity in systemic lupus erythematosus [47]. Toll-like receptors (TLRs) have been implicated in rheumatoid arthritis pathogenesis. Ang2 expression is localized in rheumatoid arthritis synovial blood vessels and TLR activation increased Ang2 expression in rheumatoid arthritis synovial extracts and endothelial cells, accompanied by increased angiogenesis [48].

### 4.2. Pneumonia

Pneumonia is a disease that affects the respiratory system and has long been recognized as a major cause of death. It is a common cause of acute respiratory distress syndrome and acute respiratory failure, it can occur as a result of infectious causes, such as bacteria and viruses, or noninfectious causes, such as pulmonary edema and lung cancer [49,50]. The pathophysiology of pneumonia and acute respiratory distress syndrome involves the induction of pulmonary endothelial permeability [51,52]. The role of Ang2 in pneumonia was demonstrated in a study comparing the serum levels of Ang2 of pneumonia patients. Ang2 levels of pneumonia patients were higher compared with healthy individuals, and the highest levels were identified in patients with community-acquired pneumonia who died within 28 days of diagnosis, compared to survivors. Furthermore, pneumolysin stimulation increased Ang2 release and ventilated and perfused lungs of mice with Ang2 knockdown presented reduced permeability on pneumolysin stimulation, whereas Ang1 therapy reduced permeability in murine pneumonia [53].

### 4.3. Mycoplasma Pulmonis Infection

In murine respiratory mycoplasmosis, an infection characterized with inflammatory-like symptoms, Ang2 protein expression was increased, contrary to Ang1 expression, and Tie2 phosphorylation in the mucosal blood vessels was decreased, indicating decreased Tie2 signaling. Ang2 blockade reduced vascular remodeling, leukocyte influx, and infection severity, supporting the inflammatory role of Ang2 [54].

### 4.4. Sepsis

Aberrant vascular leakage is the major characteristic of septic conditions. Ang2 has been associated with sepsis-related conditions [55,56,57,58,59,60] and its levels are proportional to sepsis severity [57,60]. Ang2 levels are also inversely associated with nitric oxide bioavailability. Nitric oxide inhibits Weibel-Palade body exocytosis, where Ang2 is stored, and its levels are decreased in sepsis [60]. Increased Ang2 levels have been related to high mortality rates, the number of organ failure, and the development of acute respiratory distress syndrome [56,57,58]. Ang2 knockdown by siRNA blocked neutrophil infiltration and vascular leakage, ameliorating the severity of the condition and improving survival [59]. Along with heparin-binding protein and procalcitonin, Ang2 has been recently suggested as a robust predictor of severe sepsis during the early stages of infection and prior to the appearance of the clinical symptoms [55]. Indirect acute respiratory distress syndrome (iARDS) is a life-threatening condition characterized by the loss of endothelial cell barrier function and can lead to hemorrhagic shock and sepsis. Elevated levels of Ang2 were consistent with the prognosis of the disease and silencing Ang2 reduced the occurrence of hemorrhagic shock and septic events [61], and plasma Ang2 has been reported as a potential causal marker for sepsis-associated ARDS [62]. The increased Ang2 levels in severe sepsis were also confirmed in another clinical study, and correlations with serum tumor necrosis factor-alpha and interleukin-6 were identified. However, treatment of human microvascular endothelial cells with these inflammatory mediators led to a reduction of Ang2 levels, demonstrating that the lung endothelium may not be responsible for Ang2 release in human sepsis [63].

As mentioned above, sepsis, like other inflammatory conditions, involves induction of endothelial permeability and is characterized by an accumulation of inflammatory cells, such as neutrophils. To initiate inflammation, neutrophils need to adhere to the vascular intima. This adhesion is initiated by the tethering of L-, P-, and E-selectins and α4 integrins to ligands that are located on the endothelial cell surface [64,65]. The surface of the vascular intima is coated by a gel-like endothelial glycocalyx layer, consisting of membrane-bound macromolecules, such as proteoglycans, glycosaminoglycans, glycoproteins, and plasma proteins [64]. A study conducted in sepsis patients after major abdominal surgery showed elevated levels of glycocalyx markers in the plasma of these patients compared to the ones undergoing major abdominal surgery, but had no sepsis, demonstrating a flaking of the endothelial glycocalyx in sepsis [66]. The importance of the endothelial glycocalyx in this condition was explicated in an elegant study demonstrating that sepsis induced heparanase activation in the pulmonary microvasculature, leading to degradation of the pulmonary endothelial glycocalyx, thus aiding neutrophil adherence and inflammatory lung injury [67]. Due to its role in vascular leakage in vitro and in vivo [16,68], the effect of Ang2 on endothelial glycocalyx was investigated. Ang2 was found to reduce the thickness of the endothelial glycocalyx to the same level as heparinase I and this occurred through a Tie2- and heparanase-dependent manner in endothelial cells. This was also demonstrated in vivo, where through the use of the Miles assay, the authors demonstrated that Ang2 induced the flaking of the endothelial glycocalyx [69].

### 4.5. Cancer

Studies on tumor angiogenesis and metastasis have been able to emphasize the importance of Ang2 in cancer, and for this, Ang2 has also been proposed as a biomarker in different cancer types (Figure 1). In principle, Ang2 expression levels are proportional to the cancer stage for both small and non-small cell lung cancers. However, further studies are required to identify the exact role and expression levels in each type and subtype of lung cancer [70]. The great outcome that Ang2 blockade had in xenograft models prompted the initiation of clinical trials targeting Ang2 as a monotherapy or in combination with other pathway inhibitors, such as VEGFR signaling pathway inhibitors [18,39,71]. The synergistic effect between Ang2 and VEGF was demonstrated in hepatocellular and endometrial carcinoma-induced angiogenesis [72,73].

The bottleneck in cancer research is cancer metastasis. Both Ang2 and VEGF are potent inducers of permeability, therefore co-inhibition of both growth factors yielded greater reduction of lesion permeability and reduced brain metastases in breast cancer models than inhibition of each one individually [74]. Endothelial-specific Ang2 overexpression increased the metastatic burden in lymph nodes and lungs, whereas administration of Ang2-blocking antibodies, apart from blocking lymphangiogenesis, also inhibited lymph node and lung metastasis and tumor cell homing to the lungs [38]. In a population study conducted among liver cancer patients, Ang2 was negatively correlated with overall survival. Ang2 levels were up-regulated in the liver when compared to normal tissue and these patients generally had higher levels of Ang2 than the healthy population. After surgery, Ang2 levels were observed to decline [75]. The tumorigenic and angiogenic activity of Ang2 could also be due to its effect on Tie2-expressing macrophages. Ang2 inhibition decreased the upregulation of Tie2 in these macrophages, along with their association with vasculature and angiogenic potential in metastatic MMTV-PyMT mammary carcinomas and RIP1-Tag2 pancreatic insulinomas [36]. miRs play a major role in cancer progression, and pancreatic cancer, one of the most aggressive cancers, is not an exception [76,77]. miR-145 is a known tumor suppressor in pancreatic cancer and its anti-cancer activity was attributed to inhibition of Ang2 expression. Ang2 was a direct target of miR-145, as miR-145 overexpression downregulated Ang2 in the BxPC3, MiaPaCa-2, and Panc-1 cells, suppressing cell invasion and colony formation, and inhibiting the growth of pancreatic cancer xenografts and angiogenesis in vivo [78]. EMT is the reprogramming process the tumor cells have to facilitate in order to metastasize [79]. Ang2 was shown to induce EMT in cancer. Ang2 expression was increased in lung cancer cell lines and its knockdown inhibited the EMT process, promoting E-cadherin expression and down-regulating vimentin, Twist, and Snail expression. Similarly, blockade of Ang2 expression in cervical cancer cells decreased vimentin expression and micro-vessel density, demonstrating its role on EMT and eventually cancer metastasis [80,81].

Kaposi’s sarcoma is a malignancy of endothelial origin, often found in HIV patients. Ang2, Tie1, and Tie2 receptors are highly expressed in Kaposi’s sarcoma, indicating that they play a vital role in the pathophysiology of the disease [82,83]. Both blood and lymphatic endothelial cells are susceptible to infection by Kaposi sarcoma herpesvirus (KSHV), which induces transcriptional reprogramming towards the lymphatic endothelial phenotype [84]. KSHV infection leads to Ang2 overexpression and also induces the rapid release of stored Ang2 molecules from the Weibel-Palade bodies [85], explaining the elevated Ang2 serum levels in Kaposi’s sarcoma patients [84]. Inhibition of Ang2 levels after KSHV infection inhibited angiogenesis and tumor growth and reduced the number of infiltrating immune cells [19].

Pericytes play an important role during tumor angiogenesis and affect the metastatic burden. It was observed that pericyte depletion in hypoxic tumors led to increased vascular leakiness and higher metastatic rate. Tumor transcriptomic analysis after pericyte depletion revealed increased *Ang2* transcription in endothelial cells and a 3-fold increase of Ang2 protein levels in tumors. Ang2 inhibition restored vascular stability and decreased the metastatic potential of the pericyte-depleted mice [86].

Multiple myeloma is a hematological malignancy. Patients with active multiple myeloma presented high Ang2 levels in their serum. Ang2 was produced by multiple myeloma endothelial cells, affected endothelial cell functions through Tie2 phosphorylation, and its concentration correlated with bone marrow micro-vessel density [87].

### 4.6. Cardiovascular Disease

Ang2 is involved in endothelial physiology and cardiovascular remodeling. Endothelial dysfunction is associated with cardiovascular risk factors and cardiovascular remodeling entails the alteration of the vascular structure, a precursor of cardiovascular disease [88,89,90]. Elevated Ang2 levels have been observed in most cardiovascular disorders, such as coronary heart disease [91,92,93], congestive heart failure [94], and peripheral arterial disease [92], and in associated conditions, such as chronic kidney disease [95], in most of which, Ang2 has been reported as biomarker. Ang2 also seems to play an important role in post-stroke recovery. In the first 3 days after stroke, Ang2 levels are increased, which is associated with deleterious vascular permeability, while high Ang2 levels after day 7 are correlated with microvessel stabilization and maturation [96]. Post-stroke Ang2 upregulation was reported in the subventricular zone, where Ang2 promoted neural progenitor cell differentiation and mediated their migration through a Tie2-independent manner [97]. On the other hand, decreased Ang2 expression, combined with increased Ang1 expression, is considered to contribute to vascular remodeling and this was targeted through human umbilical cord mesenchymal stem cell-conditioned medium post-stroke treatment [98]. Ang2-targeted treatment in cardiac allografts was able to prevent ischemia-reperfusion injury and chronic rejection by inhibiting endothelial cell activation and leukocyte infiltration [99].

### 4.7. Diabetic Retinopathy

Vascular leakage is one of the characteristics of diabetic retinopathy, which can lead to macular edema and vision loss. Serum Ang2 levels were also found significantly elevated in type 2 diabetic patients with both non-proliferative and proliferative diabetic retinopathy, compared to diabetic patients without retinopathy [100]. In the early streptozotocin-induced diabetic retinopathy model, an increase in Ang2 levels led to astrocyte loss and vascular leakage, both of which were blocked by intravitreal injection of an Ang2-neutralizing antibody [101]. Diabetic retinopathy is also characterized by pericyte loss. Hyperglycemia induces *Ang2* transcription, which then leads to apoptosis and migration of retinal pericytes through Tie2 activation, thus regulating pericyte activation and death. This was not observed in Ang2-deficient mice, demonstrating the impact of Ang2 on diabetic retinopathy and the potential of Ang2 inhibition for therapeutic intervention [102,103].

### 4.8. Obesity

Ang2 has been shown to play a role in adipose tissue physiology. Adipose tissue-specific Ang2 overexpression was more evident in the subcutaneous white adipose tissue and mice overexpressing Ang2 exhibited better vascularization and reduced inflammatory changes, leading to healthier adipose tissue expansion, resistance to weight gain due to a high fat diet, improved metabolic functions, and enhanced glucose tolerance, insulin sensitivity, and disposal. Ang2 blocking led to decreased vascular density in the subcutaneous white adipose tissue, characteristic of unhealthy adipose tissue expansion [104].

### 4.9. Bone Wound Healing

Since Ang2 has an effect on angiogenesis, it was expected it would play a role in bone physiology as well. Ang2 is involved in the angiogenesis associated with novel bone formation and Ang2 expression has been correlated with the speed of bone healing [105]. During forward mandibular positioning in the condylar chondrocytes of rabbits, Ang2 expression in the condylar cartilage was increased, concomitant with its angiogenic role during endochondral ossification [106]. Shortly after traumatic spinal cord injury, plasma Ang2 levels are increased, as part of the endogenous regenerative response; however, the mechanism is not yet clear [107]. During intervertebral disc degeneration, Ang2 expression is increased proportionally with the severity of the condition. Exogenous Ang2 administration led to the production of catabolic proteases and a decrease of aggrecan and collagen II levels, highlighting Ang2 as a therapeutic target for intervertebral disc degeneration [108]. In the bone marrow, high concentrations of Ang2 enhanced autophagy and promoted vascularization and bone defect repair on a hydroxyapatite/collagen scaffold in rabbits [109]. Similar to VEGF, the plasma Ang2 concentration is dependent on the Ang2 concentration in the bone marrow. For instance, plasma Ang2 levels in leukemia are higher than the ones in solid tumors and leukemia patients with higher Ang2 levels had longer event-free survival rates [110].

## 5. Ang2-Induced Molecular Mechanisms

The major receptor in the angiopoietin/Tie signaling pathway is Tie2. Interaction with Tie2 and Tie2 phosphorylation was initially considered the pathway solely responsible for Ang2-induced biological activity. However, recent studies revealed that Tie2 is not always responsible for Ang2-induced functions, and an interaction with other transmembrane molecules has been identified. Therefore, the molecular pathways summarized here are categorized as Tie2-dependent or Tie2-independent, based on the presence and activation of the Tie2 receptor.

### 5.1. Tie2-Dependent Signaling

**Figure 2:**
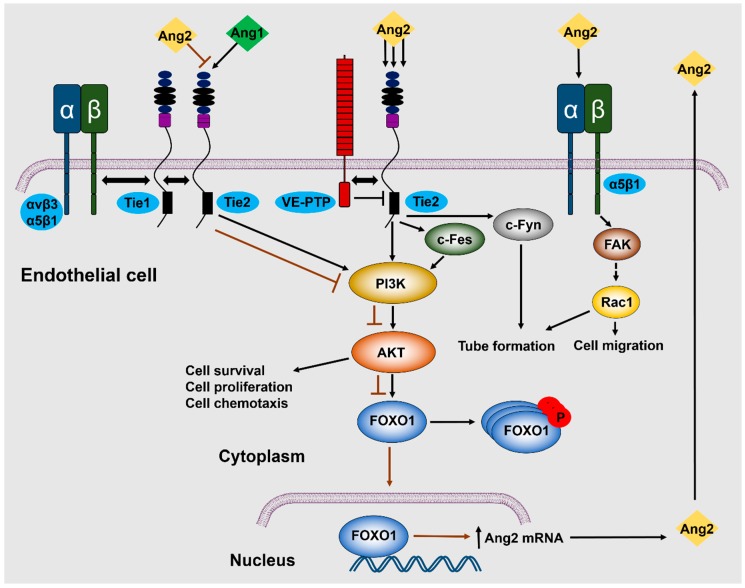
Illustration of the main signaling pathways activated upon Ang2 binding to endothelial cells. Ang2 can interact with Tie2 either as an antagonist or as an agonist, when present at high concentrations. Activation of the Tie2 downstream pathway, which occurs in the absence of VE-PTP, eventually leads to Foxo1 inhibition and upregulation of endothelial functions. Upon Ang2 binding, Tie2 heterodimerizes with Tie1 and they can both form complexes with integrins. Ang2 can bind to integrins also independently of Tie2, inducing endothelial cell migration and sprout formation.

Tie2 receptor, a tyrosine kinase receptor containing epidermal growth factor homology motifs, immunoglobulin-like loops, and fibronectin type III repeats, is expressed in endothelial [111] and hematopoietic stem cells [112] and binds with all four angiopoietins [1,3,113]. It was initially reported that Ang2 binds, but does not activate Tie2, but instead acts as a competitive Ang1 antagonist on endothelial cells [3]. A subsequent study demonstrated that at high concentrations (800 ng/mL) Ang2 can induce phosphorylation of the Tie2 receptor, leading to activation of the p85 subunit of PI3K, and Akt phosphorylation on Ser473, finally promoting cell survival and proliferation. These effects, however, were not observed at lower concentrations [21] (Figure 2). In a study where trophoblasts were observed to express Tie2 receptors, incubation with 250 ng/mL of Ang2 stimulated trophoblast proliferation and triggered nitric oxide (NO) release [29], although the pathway is still not known.

Forkhead box O (Foxo) are transcription factors regulating cell growth, development, and metabolism. Foxo1 is predominantly expressed in endothelial cells and is a negative regulator of angiogenesis [114]. Ang1, a potent angiogenesis inducer, inhibits Foxo1 through Akt activation. Akt-induced phosphorylation of Foxo1 leads to its inhibition and cytoplasmic localization, therefore repressing Ang2 expression, as confirmed by *Foxo1* knockdown experiments. PI3K inhibition blocked Akt activation, inhibiting Foxo1 phosphorylation, inducing its nuclear accumulation and thus activation, leading to a significant increase in Ang2 mRNA levels. The produced Ang2 acted in an autocrine manner, phosphorylating Tie2, activating Akt, and thus inhibiting, to a certain extent, but not completely, Foxo1 activation and Foxo1-mediated transcription and apoptosis [114,115,116] (Figure 2).

Ang2-induced Tie2 phosphorylation led to chemotaxis and tube formation of murine capillary brain endothelial cells. Chemotaxis was mediated by PI3K through c-Fes activation, whereas tube formation was independent of PI3K activation and was mediated by c-Fyn activation [117]. It was noted that the orphan receptor, Tie1, participates in the regulation of angiogenesis by Ang1 and Ang2: Ang1 and Ang2 binding to Tie2 leads to Tie1–Tie2 interaction through β1 integrin, while Tie1 cleavage leads to a reduction and loss of Ang1 and Ang2 agonist activity, respectively, through inhibited Tie2, AKT phosphorylation, and increased Foxo1 activation [14]. In another study, it was reported that both Tie1 and Tie2 can bind the integrins, α5β1 and αvβ3, and this interaction was enhanced in the presence of fibronectin. Contrary to Ang1, the Ang2 receptor binding domain (the fibrinogen-like domain that does not include the coiled coil and super clustering domains of the ligands) could not directly interact with either integrin independently of Tie2 [118]. Ang2 stimulation induced a complex formation between Tie2, αvβ3 integrin, and focal adhesion kinase (FAK), leading to FAK phosphorylation on Ser910, αvβ3 integrin internalization, and endothelial cell destabilization [119].

Tie2 activation is not solely facilitated by Ang1 or Ang2, but can be activated by other growth factors or cleaved Tie2 molecules. VEGF was shown to activate Tie2 in HUVECs, without direct interaction, but through proteolytic cleavage of Tie1, leading to Tie2 trans-phosphorylation. This process was mediated by metalloproteinases, as Tie2 activation was blocked with the metalloproteinase inhibitor, TAPI-2 [120]. The Tie2 ectodomain has been reported to be released from the cell surface through protease cleavage (receptor shedding). HUVEC supernatant and human serum present a soluble form of Tie2 (sTie2), which can bind both Ang1 and Ang2 and inhibit Ang1- and Ang2-mediated Tie2 phosphorylation and the downstream biological activity. sTie2 production was induced by PMA and VEGF, through the phosphoinositide 3-kinase/Akt pathway [121]. VE-cadherin is an important mediator of the endothelial barrier [122]. Ang2 affects VE-cadherin phosphorylation, regulating its activity. VE-cadherin phosphorylation at tyrosine residue 685 induces permeability due to VE-cadherin destabilization and dysfunctional cell–cell junctions. Inhibition of Ang2 in the lymphatic endothelial cells blocked this phosphorylation, leading to the formation of button-like junctions in initial lymphatics which impaired lymph uptake and vessel leakage due to disrupted adherens junctions in collecting lymphatics [123].

### 5.2. Tie2-Independent Signaling

Since Tie2 is the main receptor of the angiopoietin/Tie signaling pathway, it was expected that Ang2-triggered molecular pathways would be solely regulated by Tie2. However, there are studies demonstrating Ang2-induced activation of Tie2-independent signaling pathways. Initially, Mochizuki et al. reported that Ang2-mediated PI3K activation was independent of the association of PI3K to Tie2 [117]. However, the notion of Tie2-independent signaling became apparent after the striking finding that integrins are alternative receptors for Ang2. In that study, it was reported that Ang2-induced cell migration and sprouting angiogenesis through FAK phosphorylation and Rac1 activation took place in Tie2-negative endothelial cells [17] (Figure 2). The integrin identified as a potent Ang2 receptor was β1 integrin. Ang2 was found to specifically bind to β1 integrin and destabilize the endothelium in a Tie2-independent manner [124]. Moreover, β1 integrin binding and activation was Ang2-specific, but not Ang1, and expression of a membrane-bound Tie2 lacking the kinase activity blocked the activation of the integrins by Ang2 [124]. Apart from the β1, the α5 subunit of α5β1 integrin is also important for Ang2–α5β1 interaction, as it was shown to interact with Gln-362 of Ang2, regulating cell migration in a Tie2-independent manner [125]. Silencing of Ang2, β1 integrin, or α5-integrin inhibited endothelial monolayer destabilization by thrombin, interleukin (IL)-1β, or LPS in an endotoxemia model, further highlighting the significance of β1 integrin in Ang2-induced endothelial cell functions [126]. Ang2 interaction with integrins also takes place in the presence of Tie2, as it was shown and mentioned before that Ang2 induces a complex between β3 integrin and Tie2 in cell–cell junctions. Ang2 treatment induced FAK phosphorylation at Ser910, leading to internalization and lysosomal degradation of αvβ3 integrin [119]. Finally, direct binding of Ang2 with integrin has also been observed in astrocytes, where Ang2 directly interacted with αvβ5 integrin, leading to GSK-3β/β-catenin-induced astrocyte apoptosis and increased vascular leakage in early streptozotocin-induced diabetic retinopathy [101].

## 6. Therapeutic Strategies

The involvement of the Ang/Tie system in the pathophysiology of several disorders has rendered Ang2 as a potential therapeutic target and some therapeutic approaches also targeting Ang2 have provided promising results. Trebananib is a peptide-Fc fusion protein, which inhibits angiogenesis by blocking the binding of both Ang1 and Ang2 to the Tie2 receptor. In a randomized, double-blind, placebo-controlled phase 3 trial for recurrent ovarian cancer, Trebananib in combination with paclitaxel inhibited angiogenesis, leading to prolongation of progression-free survival [127], while in a similar study, Trebananib combined with pegylated liposomal doxorubicin also demonstrated anticancer activity, however, it failed to increase progression-free survival [128]. MEDI3617, a selective Ang2 inhibitor, was tested as monotherapy or combined with bevacizumab or cytotoxic chemotherapy in a phase I study of ovarian cancer and glioma and although it was well tolerated, it had limited clinical activity and was discontinued [129]. Vasculotide is an Ang-based peptidomimetic compound, which binds the Tie2 receptor and elicits downstream signaling, promoted angiogenesis in vitro and in vivo and accelerated wound healing in diabetic mice [130]. Vasculotide was also successful in protecting the endothelial monolayer from sepsis-associated permeability, blocking vascular leakiness, and protecting from LPS-induced and polymicrobial abdominal sepsis-induced lethality [131,132]. Inhibition of vascular leakiness was verified in other models, where it preserved microvascular integrity during hemorrhagic shock and cardiopulmonary bypass [133,134], and was effective in skin-related conditions, as it protected skin from ionizing radiation damage and ameliorated atopic dermatitis symptoms [135,136].

## 7. Conclusions

Ang2 is vital for endothelial cell physiology and plays a central role in vascular-related diseases, by regulating endothelial permeability and angiogenic functions. Here, we summarized the current knowledge on Ang2-induced effects on blood and lymphatic endothelial cells, its role in vascular-related diseases, and provided a general overview of Ang2-induced signaling pathways in endothelial cells. Given the active role of Ang2 in many diseases, targeting of the Ang/Tie pathway is a promising approach, especially now that the current anti-angiogenic therapies are under serious consideration, and the existing clinical data is encouraging. Ang2 interacts with different proteins and has diverse context-dependent effects on different cell types, which have not yet been fully elucidated. Therefore, more research is required to understand the contradicting roles of Ang2 and the Ang2-induced signaling circuitries in different diseases, with the ultimate goal of developing potential therapeutic targets.
